# Training system for converting current visual information to bird’s-eye view

**DOI:** 10.3389/fpsyg.2024.1504838

**Published:** 2024-12-23

**Authors:** Kaoru Sumi, Ryo Okuyama

**Affiliations:** Faculty of Systems Information Science, Future University Hakodate, Hakodate, Japan

**Keywords:** spatial perspective transformation, virtual reality sports training, virtual reality, first-person to bird’s-eye perspective conversion, spatial cognitive ability, cognitive skill development, perceptual-cognitive training, team sports decision-making

## Abstract

**Introduction:**

Effective decision-making in ball games requires the ability to convert positional information from a first-person perspective into a bird’s-eye view. To address this need, we developed a virtual reality (VR)-based training system designed to enhance spatial cognition.

**Methods:**

Using a head-mounted virtual reality display, participants engaged in tasks where they tracked multiple moving objects in a virtual space and reproduced their positions from a bird’s-eye perspective. The system simulated game-like scenarios to systematically train this cognitive skill. Participants’ performance was assessed before and after the training.

**Results:**

The experimental results revealed significant improvements in spatial accuracy and cognitive ability among participants after using the system. These enhancements were measured by their ability to accurately convert first-person positional data into a bird’s-eye perspective.

**Discussion:**

The findings suggest that the VR-based system effectively enhances perceptual-cognitive skills critical for team sports and other tasks requiring advanced spatial awareness. This training method holds potential for broader applications in spatially demanding activities.

## 1 Introduction

In ball games, which are sports that use a ball, top players are often distinguished by their ability to better grasp the situation and quickly convert a first-person viewpoint into a bird’s-eye view. Ball games such as soccer, handball, and American football involve scoring by passing the ball into a goal. These games are typically played by large teams, making it crucial for players to understand the positions of their teammates and opponents. This ability to quickly perceive and process spatial relationships is essential for strategic gameplay (Mashimo, 2002; Mann et al., 2007; Williams et al., 2004).

However, from a first-person perspective, it is difficult to perceive distances between players and identify open spaces because other players often obstruct the view. Additionally, decisions must be made in split seconds. Research has shown that players with strong bird’s-eye perspective skills can better understand the broader game situation, enabling them to make faster and more accurate decisions (Fujii et al., 2014; Shepard and Metzler, 1971). Thinking from a bird’s-eye view—organizing information spatially from that perspective—allows players to make accurate, informed decisions during a game. Skilled players are adept at this, enabling them to understand the broader situation rather than relying on localized information, which leads to superior situational judgment.

For example, Xavi Hernández, a renowned player for Spain’s national soccer team and FC Barcelona, was featured on NHK’s Special Miracle Body, a Japanese TV program, for his exceptional bird’s-eye perception. In the 2013 FIFA Confederations Cup, he reportedly moved his head more than 850 times per match, suggesting that understanding one’s surroundings is critical in games and that continuous scanning of the environment is key.

Developing the ability to convert first-person information into a bird’s-eye view requires extensive game experience. Unlike individual skills, such as shooting practice, this type of training demands the cooperation of many players, posing logistical challenges. It is difficult for a single player to practice this skill independently due to the need for both a large space and many participants. Furthermore, there is no real-world situation where a first-person view can be instantly switched to a bird’s-eye view, and thus, no established training methods exist.

Recent research underscores the potential of virtual reality (VR) in addressing these challenges by creating controlled environments for training spatial cognition and bird’s-eye view abilities. Shimizu and Sumi (2017) developed a training system where users relived first-person perspectives to improve situational judgment in ball games. They demonstrated that users could consciously improve their positioning and decision-making through VR-based scenarios. Expanding on this, Shimizu and Sumi (2019) introduced a system enabling users to simulate ball games in 360° first-person views and rearrange players and balls from a bird’s-eye perspective. These approaches illustrate the utility of VR in connecting first-person actions to spatial arrangements, making it a powerful tool for sports training.

Further, Nuruki et al. (2023) assessed and trained football players’ ability to reconstruct player positions from a bird’s-eye view using VR, finding that experienced players outperformed novices in positional accuracy. Similarly, Miura et al. (2023) employed drones for real-time feedback to enhance spatial positioning in baseball scenarios. Kanda et al. (2023) investigated VR-based training effects on visual search behaviors in soccer, revealing that VR can improve perceptual strategies for bird’s-eye view cognition. These findings collectively highlight the growing relevance of VR in systematically improving spatial cognition and situational awareness in team sports.

In this study, we focus on developing a training system to improve the ability to convert first-person information into a bird’s-eye perspective, utilizing virtual reality technology and a Head Mounted Display (HMD). This system allows individual players to train without requiring large numbers of people or large spaces, as it replicates game-like conditions in a virtual space.

Vision is known to play a critical role in ball games, and vision training has been implemented in the past. Research has shown that high-level athletes often possess superior perceptual-cognitive abilities (Mann et al., 2007; Morris-Binelli and Müller, 2017). One example is the Three-Dimensional Multiple Object Tracking (3D-MOT) system from Neuro Innovation Corporation, known commercially as NeuroTracker, is widely used by athletes for training to enhance perceptual-cognitive abilities. This system has been shown to improve decision-making and situational awareness by training users to track multiple moving objects in dynamic 3D environments (Faubert, 2013; NeuroTracker, 2024). In this task, participants track multiple moving objects in a 3D space and identify specific ones after several seconds. In a study involving 23 soccer players, 3D-MOT training was shown to improve decision-making accuracy in passing, dribbling, and shooting (Legault and Faubert, 2012). Similarly, other studies have demonstrated that perceptual-cognitive training using 3D-MOT improves on-field passing decisions (Romeas et al., 2016). Beyond sports, 3D-MOT has also been applied to reduce the effects of aging on perceptual-cognitive processes in older adults (Legault et al., 2013). However, while similar in that it tracks overlapping objects, 3D-MOT does not involve converting a first-person viewpoint to a bird’s-eye view, which is the focus of our study.

Eye-tracking studies in sports, such as those by Bard and Fleury (1976) and Kredel et al. (2023) have explored how players’ gaze behavior influences decision-making. For example, in a basketball study, skilled players gazed less frequently, focusing more efficiently on key areas. Similarly, in soccer, expert goalkeepers spend more time fixating on crucial areas during penalty tasks (Piras and Vickers, 2011). The difference between skilled and novice players is particularly evident in tasks requiring complex decision-making, as shown by Gegenfurtner et al. (2011).

Perceptual-cognitive training using video-based methods has also been shown to enhance athletes’ ability to anticipate and react. For instance, tennis players improved their ability to predict serve type and location after video-based training (Farrow et al., 1998; Larkin et al., 2016).

Virtual reality (VR) is increasingly being applied in sports training. For example, GameSense Sports offers a video occlusion product for baseball batting training (Lemire, 2017), while Trinity VR provides a system for virtual batting practice with animated pitches (Lemire, 2017). EON Sports’ VR system allows users to simulate pitchers’ throws, enabling them to train based on specific pitch characteristics (DiGiovanni, 2016). STRIVR has been used in American football to help quarterbacks practice defensive reads in realistic virtual environments, especially for backups who do not get many practice repetitions (Feldman, 2015). The system adapts to the user’s performance, presenting throws of varying difficulty to match the user’s skill level (Gray, 2017).

While various tools have been developed, it has been suggested that integrating perceptual-cognitive training into athletes’ daily routines is essential for maximizing its benefits. Research on how to embed these technologies into regular practice sessions is still needed (Fadde and Zaichkowsky, 2018).

In developing a bird’s-eye viewpoint training system using an HMD, previous studies have explored cognitive training aimed at enhancing spatial awareness (Shitamori et al., 2016; Faubert, 2013). In one study, participants used an HMD to observe a virtual court for 10 s, memorizing the positions of 10 player objects. They were then asked to identify the closest match to the observed layout from a bird’s-eye view. However, this system had limitations because the player objects were immobile, differing from real-game conditions.

In this study, we developed a system similar to 3D-MOT that allows users to train converting first-person viewpoints into bird’s-eye views. Our system enables the reproduction of player positions based on the last observed situation in the training session. We aim to verify the effectiveness of this training system in enhancing perceptual-cognitive abilities.

## 2 Training system for converting first-person perspective to bird’s-eye view

We used the Oculus Rift and Oculus Touch by Oculus Inc. to implement this system in a virtual reality (VR) environment. The use of HMDs (Head-Mounted Displays) and VR allows the system to reproduce head movements—such as head shaking—that are essential for efficiently grasping the surrounding situation. It also provides effective training by displaying necessary information in real-time (Honjo et al., 2005; Kawamura, 2002; Farrow and Abernethy, 2003).

**Figure 1:**
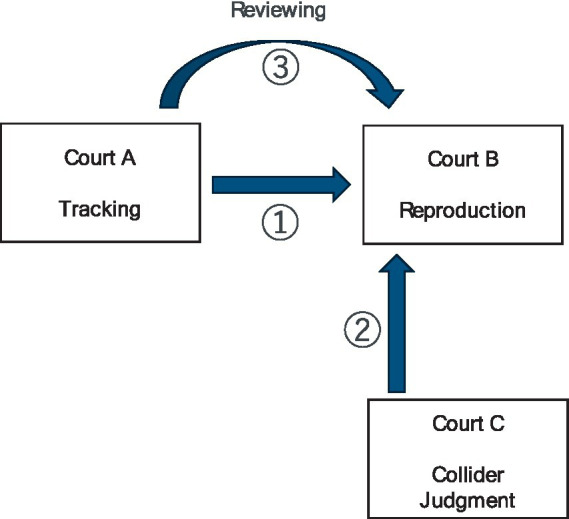
System transition diagram. The diagram represents the progression through the three phases of the training system: Tracking phase, Reproduction phase, and Review phase. During the Tracking phase, the user observes objects moving in Court A. Once the objects stop, the user transitions to Court B to recreate the observed positions from a bird’s-eye perspective in the Reproduction phase. In cases where the score is below 100, the system moves to the Review phase, enabling the user to compare their attempt with the correct positions to improve their spatial understanding. The system cycles through these phases until the user achieves a perfect score.

**Figure 2:**
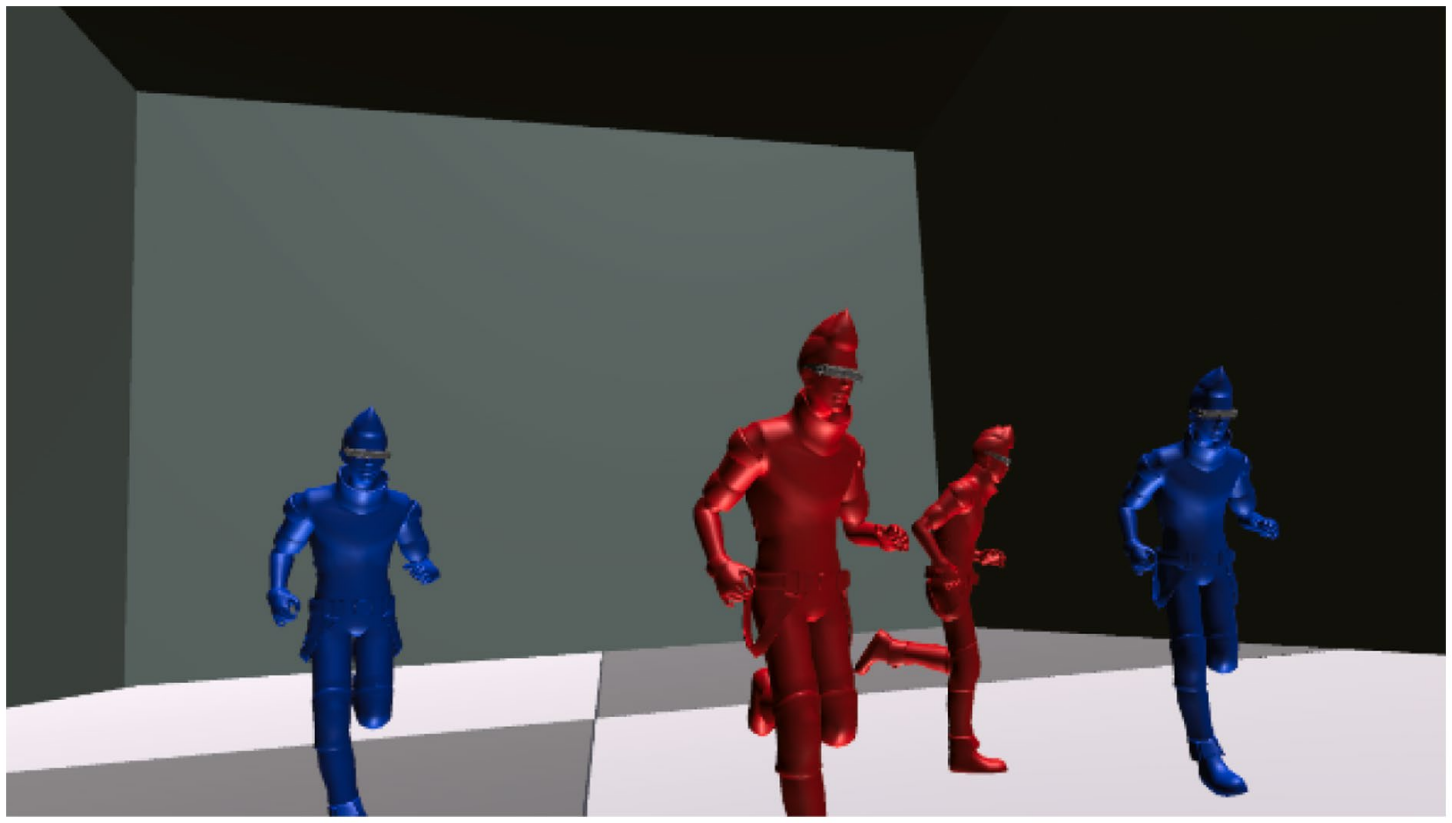
Screenshot of tracking phase in Court A. This screenshot depicts the user’s view during the Tracking phase within Court A. In this phase, the user observes and tracks multiple moving objects in a virtual environment using a Head Mounted Display (HMD). The objects move across the court in various directions, requiring the user to turn their head to follow them. This task aims to enhance the user’s ability to recognize spatial relationships and prepare for the subsequent Reproduction phase.
- Reproduction phase (Figures 3–6)

The system is divided into the following three major phases (Figure 1):

- Tracking phase (Figure 2)

**Figure 3:**
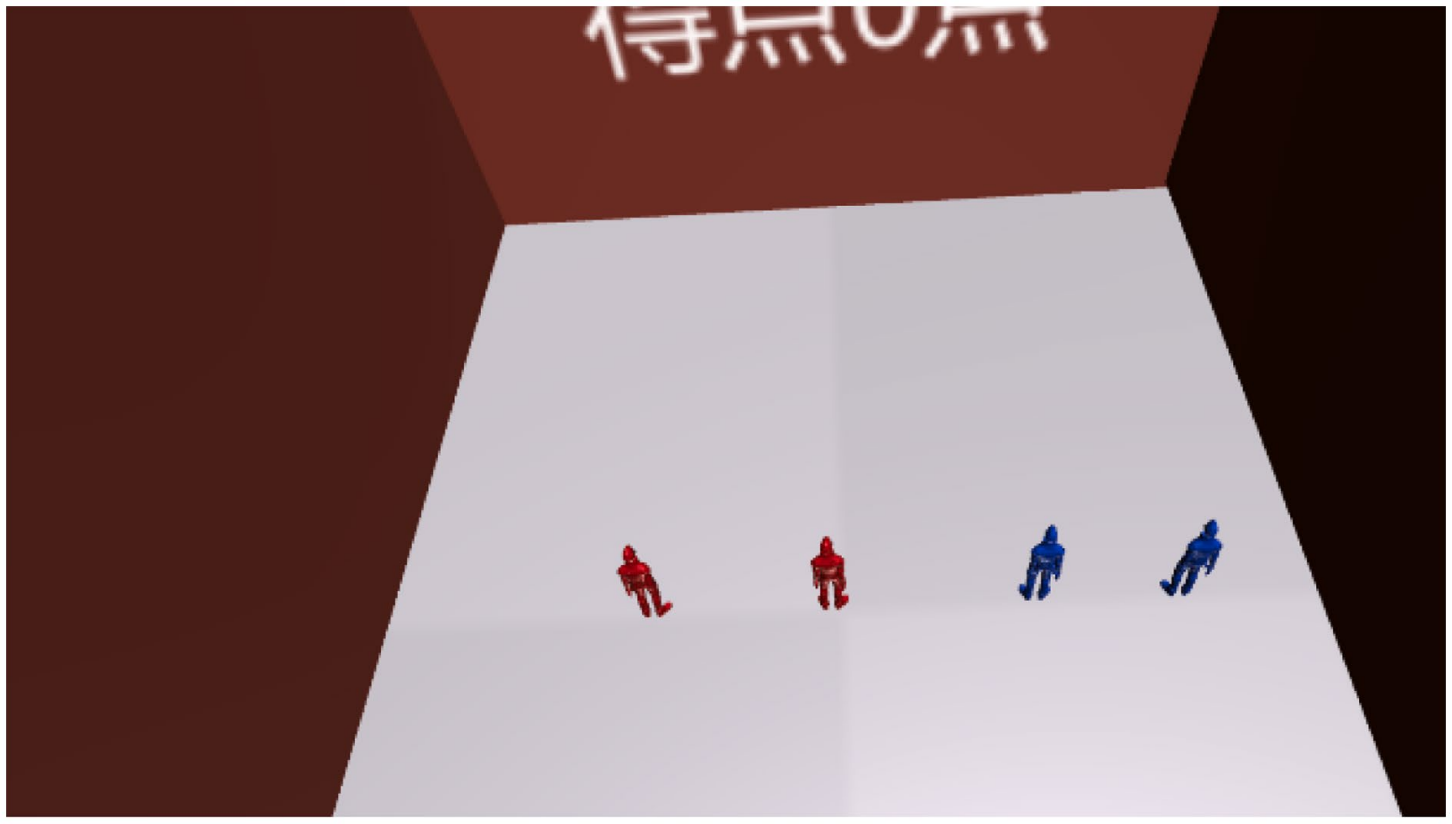
Screenshot of reproduction phase. This figure shows the Reproduction phase, where the user attempts to recreate the positions of objects observed during the Tracking phase. The user interacts with the virtual court (Court B) from a bird’s-eye view, using an HMD to place the objects in their perceived correct positions. The score is displayed after the placement to provide immediate feedback, indicating the accuracy of the user’s spatial reconstruction.

**Figure 4:**
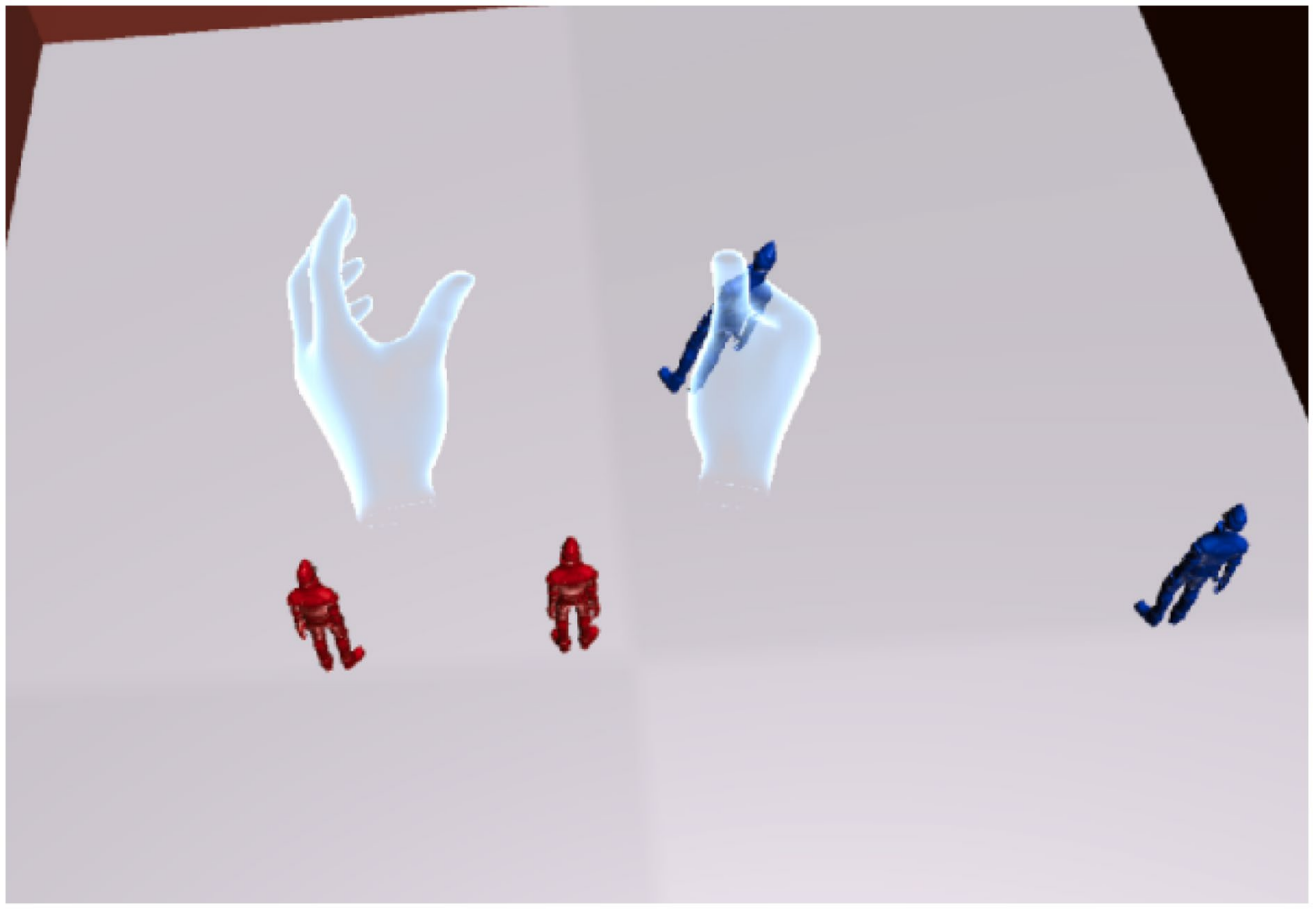
Placing objects with Oculus Touch. This figure illustrates the process of placing objects during the Reproduction phase. The user uses Oculus Touch controllers to manipulate virtual hands, allowing precise movement and placement of objects within the court. The interaction demonstrates how users adjust object positions to match their recollection of the observed layout from the Tracking phase, enabling a hands-on approach to improving spatial awareness.

**Figure 5:**
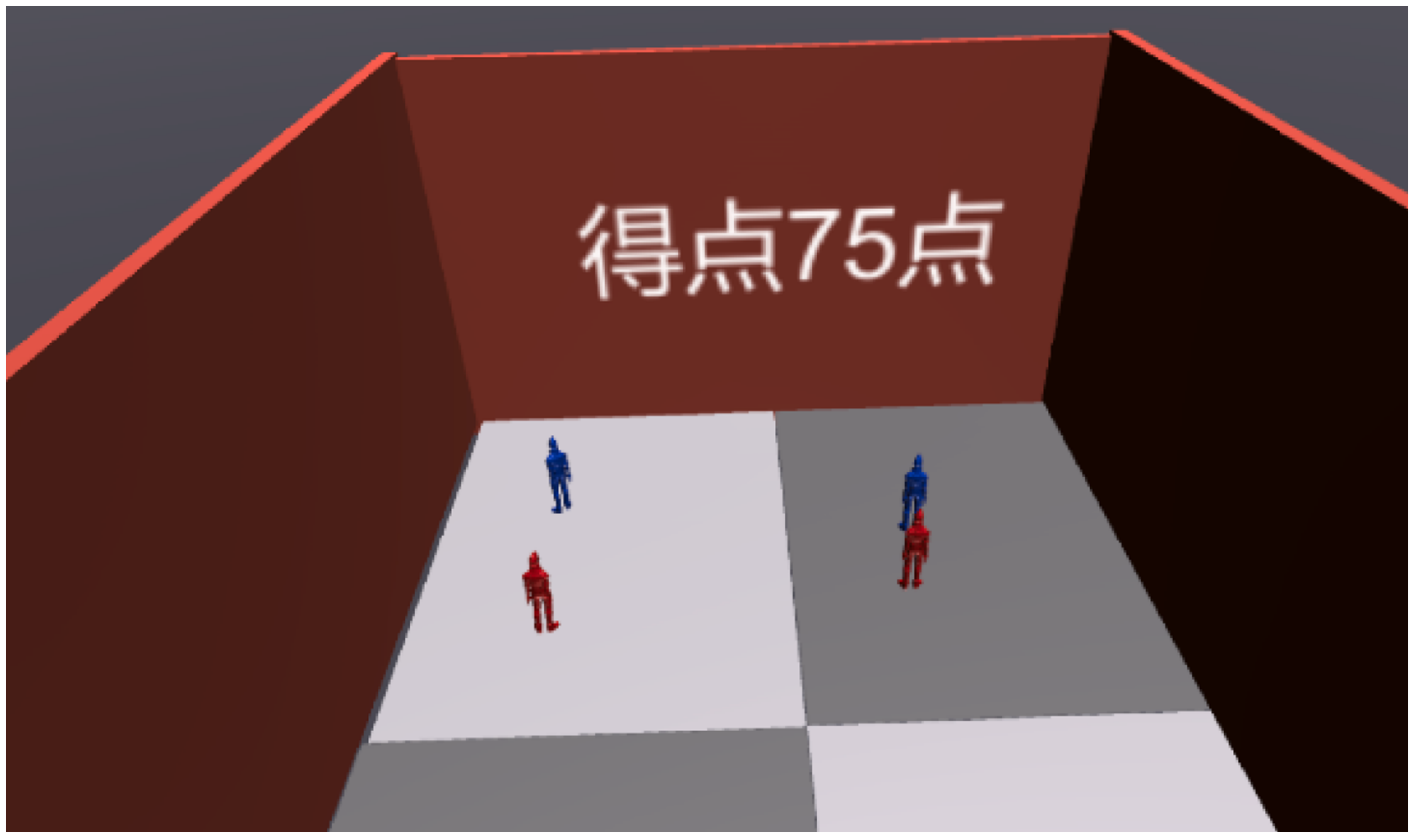
Screenshot after grading in the recall phase (75 points). This figure displays the grading results shown during the Reproduction phase after the user has placed objects in Court B. In this example, the user achieved a score of 75 points, reflecting the accuracy of their spatial reconstruction based on the observed layout in the Tracking phase. The score provides immediate feedback, allowing users to gauge their performance and identify areas for improvement.

**Figure 6:**
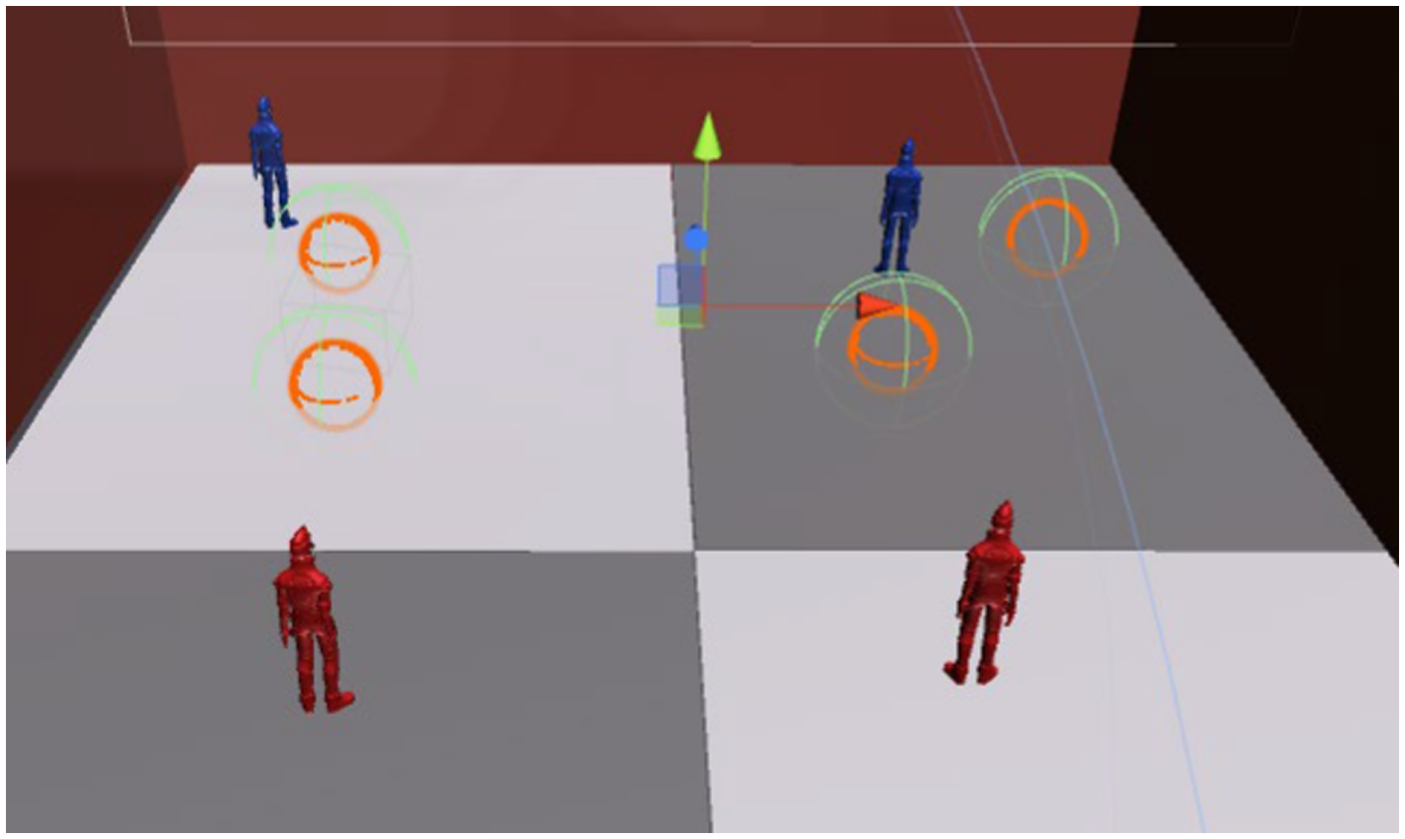
Screenshots of Court B and Court C at the time of scoring. This figure illustrates the process of scoring during the Reproduction phase, highlighting the comparison between the user’s placement in Court B and the correct layout in Court C. The system uses collision detection to determine whether the objects placed by the user overlap with the correct positions in Court C. The overlapping areas are assessed to assign points, providing an objective measure of the user’s accuracy. This mechanism ensures precise evaluation of the user’s spatial recall ability.
- Review phase (Figure 7)

**Figure 7:**
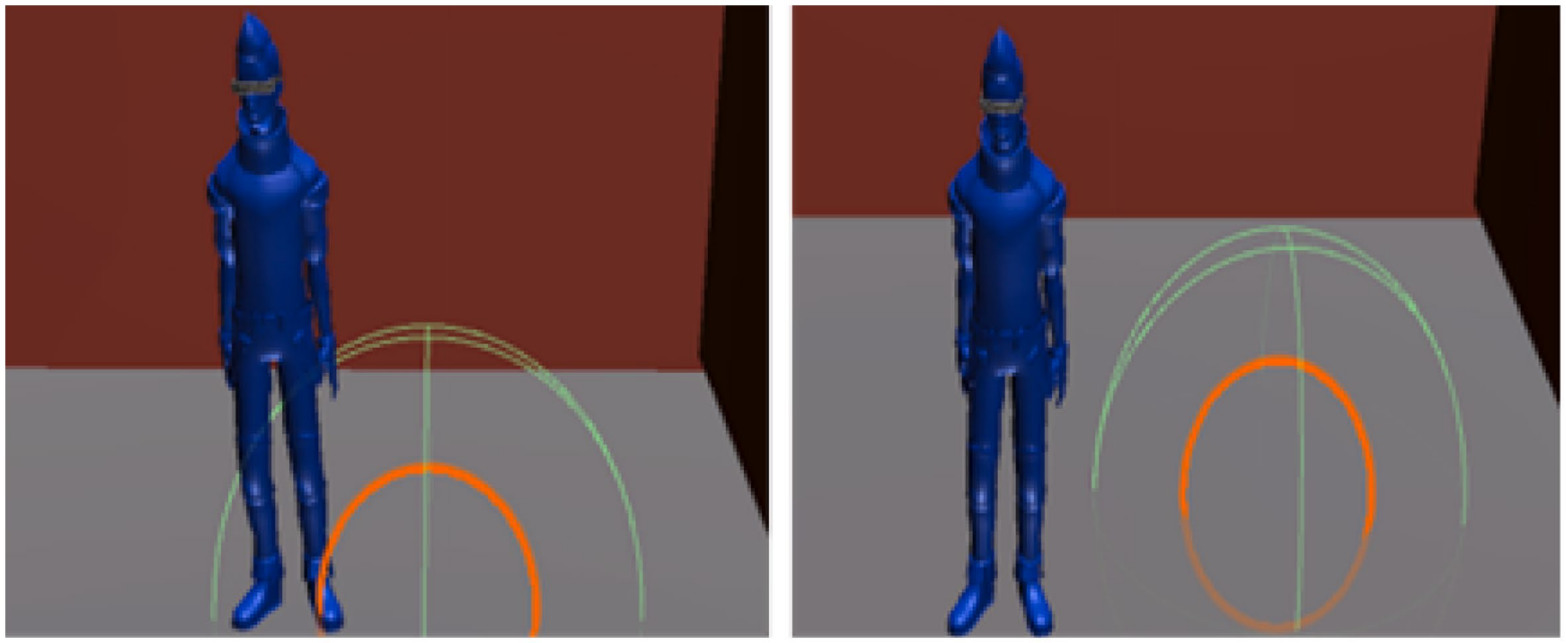
Judgment by the collider, Left is correct, right is incorrect. This figure illustrates the judgment process using the collider during the scoring phase. The system evaluates whether the user’s placement in Court B overlaps with the correct placement in Court C. The left image shows a correct placement where the object is within the collider’s range, while the right image displays an incorrect placement outside the collider’s range. This collision-based judgment forms the basis for calculating the user’s score, ensuring an objective evaluation of spatial accuracy.

### 2.1 Tracking phase

In the Tracking phase, the user is asked to track an object moving around a court in the virtual space (referred to as Court A) for several seconds. The object moves not only in front of the user but also to the sides and behind, requiring the user to shake their head to check the surroundings. As mentioned earlier, head shaking is considered important for grasping the surrounding environment. Thus, when setting the movement route of the object, we designed it to necessitate head movements. After a few seconds, the moving objects stop, and the user is asked to memorize the current situation of Court A (e.g., object positions and distances between them).

### 2.2 Reproduction phase

After the Tracking phase, the user is transferred to another virtual court dedicated to reproduction (Court B), where the viewpoint shifts to a bird’s-eye view (Figure 3). This begins the Reproduction phase. Here, the user must recreate the memorized situation from Court A from the bird’s-eye perspective. In the virtual space, the user controls virtual hands (linked to the Oculus Touch controllers) to move objects and place them in the correct positions (Figure 4). After completing the reproduction, the system proceeds to scoring (Figure 5).

During scoring, a hidden overhead court (referred to as Court C) with spheres positioned as in Court A is superimposed on Court B (Figure 6). Each sphere in Court C is assigned a collider, and a correct answer is registered when the object placed by the user falls within the collider’s range (Figure 7). The scoring system, as detailed in Table 1, evaluates participants’ performance based on the number of objects correctly placed in the bird’s-eye view reconstruction task. The system assigns points per object, with the value per correct placement decreasing as the total number of objects in the task increases. For example, tasks with 2 objects assign 50 points per correct placement, while tasks with 6 objects assign 16 points per object. Despite these variations, the system ensures that the maximum achievable score for any task is consistently capped at 100 points, maintaining a uniform evaluation scale across different configurations.

**Table 1:** Scoring system based on the number of correctly placed objects.

Number of objects	Points per object
2	50
4	25
5	20
6	16

The maximum achievable score is always capped at 100 points. For example, a score of 96 is displayed as 100. This table explains the scoring system used by the training system to evaluate participants’ performance in both the experimental and control groups. The total score is calculated based on the number of correctly placed objects in the bird’s-eye view. Each correct placement is assigned a specific point value depending on the number of objects in the test, with the system ensuring that the maximum achievable score is consistently capped at 100 points. The final score reflects the accuracy of participants in reconstructing the spatial arrangement during the tests.

The total score for each task is calculated by summing the points awarded for each correctly placed object. Additionally, a rounding mechanism is employed for display purposes; for instance, a score of 96 points is rounded up and displayed as 100, emphasizing clarity in participant feedback. These scores are then presented to participants immediately after completion (Figure 5), providing them with an understanding of their performance. This method ensures consistency in evaluation while allowing for straightforward comparisons between experimental and control groups, regardless of the task’s complexity.

### 2.3 Review phase

**Figure 8:**
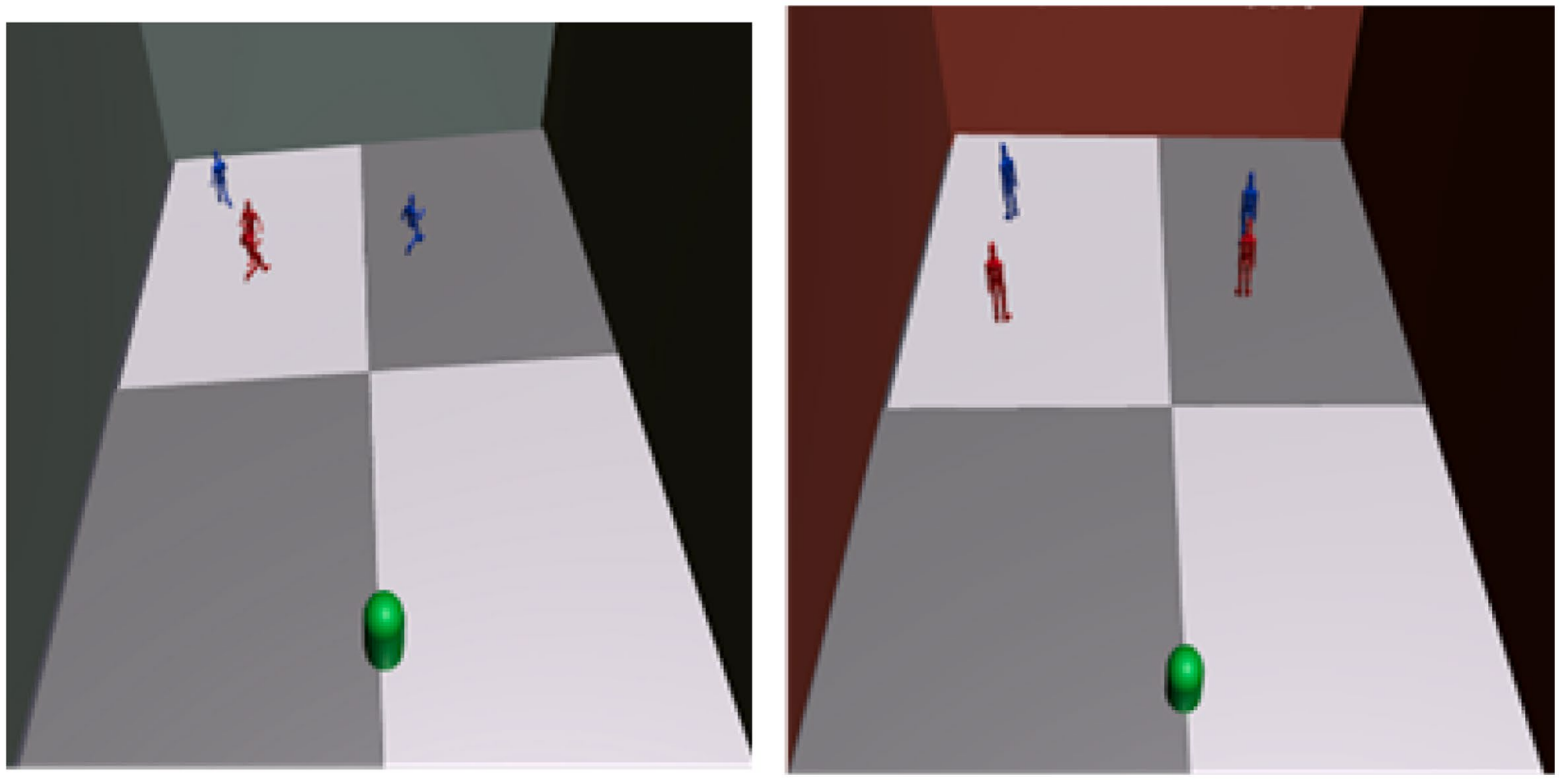
Screenshot of the review phase comparing left and right. This figure shows the Review phase, where the user can compare their object placements in Court B (right) with the correct layout observed in Court A (left). This phase alternates between the two views, allowing the user to identify discrepancies and understand where adjustments are needed. The Review phase is designed to reinforce learning by providing a visual comparison and enabling iterative improvement in spatial reconstruction skills.

If the user’s score is less than 100, the system transitions to the Review phase. In this phase, the user can observe both Court A and their reproduction of Court B from a bird’s-eye view (Figure 8). The goal is to review any mistakes by comparing the two setups, identifying which objects were misplaced. This allows the user to understand their errors and make necessary corrections until a perfect score (100) is achieved.

## 3 Experiment

The purpose of this experiment was to evaluate the effectiveness of the developed training system. Twenty-two male university students in the information field participated in the experiment. The participants were divided into two well-balanced groups of 11 students each, an experimental group and a control group, taking into account their experience with ball games. A preliminary questionnaire was administered to gather information about the subjects’ prior sports experience, and they were divided into groups to minimize differences in cognitive ability from a bird’s-eye viewpoint. To avoid bias in the number of subjects with more than 3 years of ball game experience (indicated by asterisks in the table), participants numbered 1 to 11 were assigned to the control group, and participants numbered 12 to 22 were assigned to the experimental group (Table 2).

**Table 2:** Participant demographics and sports experience.

Subjects	Sports experience (elementary, middle, and high school)
#1	Swimming 6 years, Table tennis 3 years, Handball 3 years ★
#2	Judo 4 years, Soft Tennis 3 years, None
#3	Badminton (3 months), track and field (long distance) 3 years
#4	Soccer 4 years, Football 3 years, Soccer 3 years ★
#5	None, Basketball 3 years, Handball 3 years, Handball 3 years ★
#6	Baseball 5 years, Swimming 1 year, Baseball 3 years, Ground Hockey 3 years ★
#7	Baseball 4 years, Baseball 3 years, Baseball 3 years
#8	None, None, Kyudo 3 years.
#9	Baseball 3 years, Baseball 3 years, Handball 3 years ★
#10	Baseball 2 years, Baseball 3 years, Baseball 3 years
#11	Baseball 3 years, Judo 2 years, Baseball 3 years, Handball 3 years ★
#12	Baseball 4 years, Basketball 3 years, Basketball 3 years ★
#13	Baseball 3 years, Baseball 3 years, Baseball 3 years
#14	Baseball 3 years, Basketball 3 years, Basketball 3 years ★
#15	None, None, None
#16	Table Tennis 2 years, Table Tennis 2 years, None
#17	Baseball 2 years, Hardball Tennis 3 years, None
#18	Basketball 2 years, Basketball 3 years, Basketball 3 years ★
#19	Swimming 6 years, Soccer 3 years, Basketball 3 years, Basketball 3 years ★
#20	Basketball 3 years, Basketball 3 years, Basketball 3 years ★
#21	None, None, None
#22	None, Basketball 3 years, None ★

The table provides an overview of participant demographics, including their sports experience categorized by years of engagement in ball games, to ensure balanced group assignment.

The experiment was conducted over 4 days. The experimental group completed the pre-task on day 1, followed by the training task. On days 2 and 3, they only performed the training task, and on day 4, they completed the post-task after finishing the final training task. The control group did not train with the system but completed the pre- and post-tasks on the same days as the experimental group.

**Figure 9:**
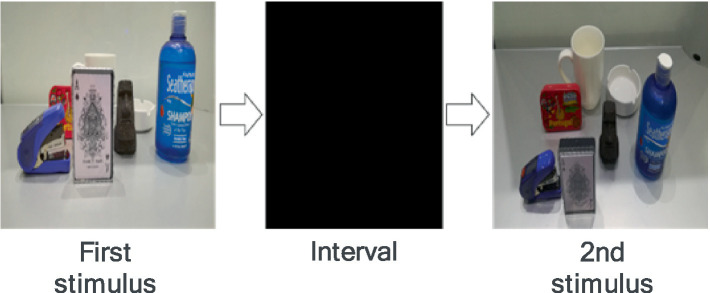
Pre-post test. This figure illustrates the sequence of the pre-post test. The test begins with the presentation of the first stimulus, showing a layout of objects observed from a first-person perspective. After a brief interval, the second stimulus is displayed, either replicating the first layout from a bird’s-eye perspective or showing a modified arrangement. Participants are required to determine whether the second stimulus correctly represents the layout from the first stimulus, testing their ability to mentally switch perspectives and reconstruct spatial relationships.

Pre- and post-tasks were designed to assess the subjects’ cognitive ability from a bird’s-eye viewpoint. The stimulus presentation method was based on Shelton and Gabrieli (2002), and the task was created with reference to Fujii et al. (2014). In the pre- and post-tasks, the first stimulus was an image of objects, such as mugs, observed from a first-person perspective. The second stimulus was either an image of the same objects presented in the first stimulus but from a bird’s-eye viewpoint or an image of the objects with a different arrangement from the bird’s-eye perspective. The first stimulus consisted of 6 or 7 randomly placed objects, shown from a first-person perspective for 1 s, followed by the second stimulus after a 1-s interval (Figure 9). The subjects were asked to judge whether the second stimulus image correctly represented the first stimulus from the bird’s-eye viewpoint. They were instructed to respond verbally with “correct” if the arrangement was the same, and “incorrect” if it was not. Before starting the task, a practice stimulus was presented with an image of four objects, and the task was explained to the participants.

The training task was conducted using the developed training system. First, the subject put on the Oculus Rift and adjusted it for optimal focus. At the beginning of each training session, participants completed a tutorial using two objects to familiarize themselves with the VR environment and to learn the flow of the training task. After completing the tutorial, we confirmed that the participants were not experiencing any issues, such as VR sickness, and then had them proceed with the actual tasks. The tasks followed the order of the Tracking phase, Reproduction phase, and Review phase. The training included two tasks with four objects, two tasks with five objects, and one task with six objects. Since most participants had no prior experience with VR, they only performed each task once on the first day.

**Figure 10:**
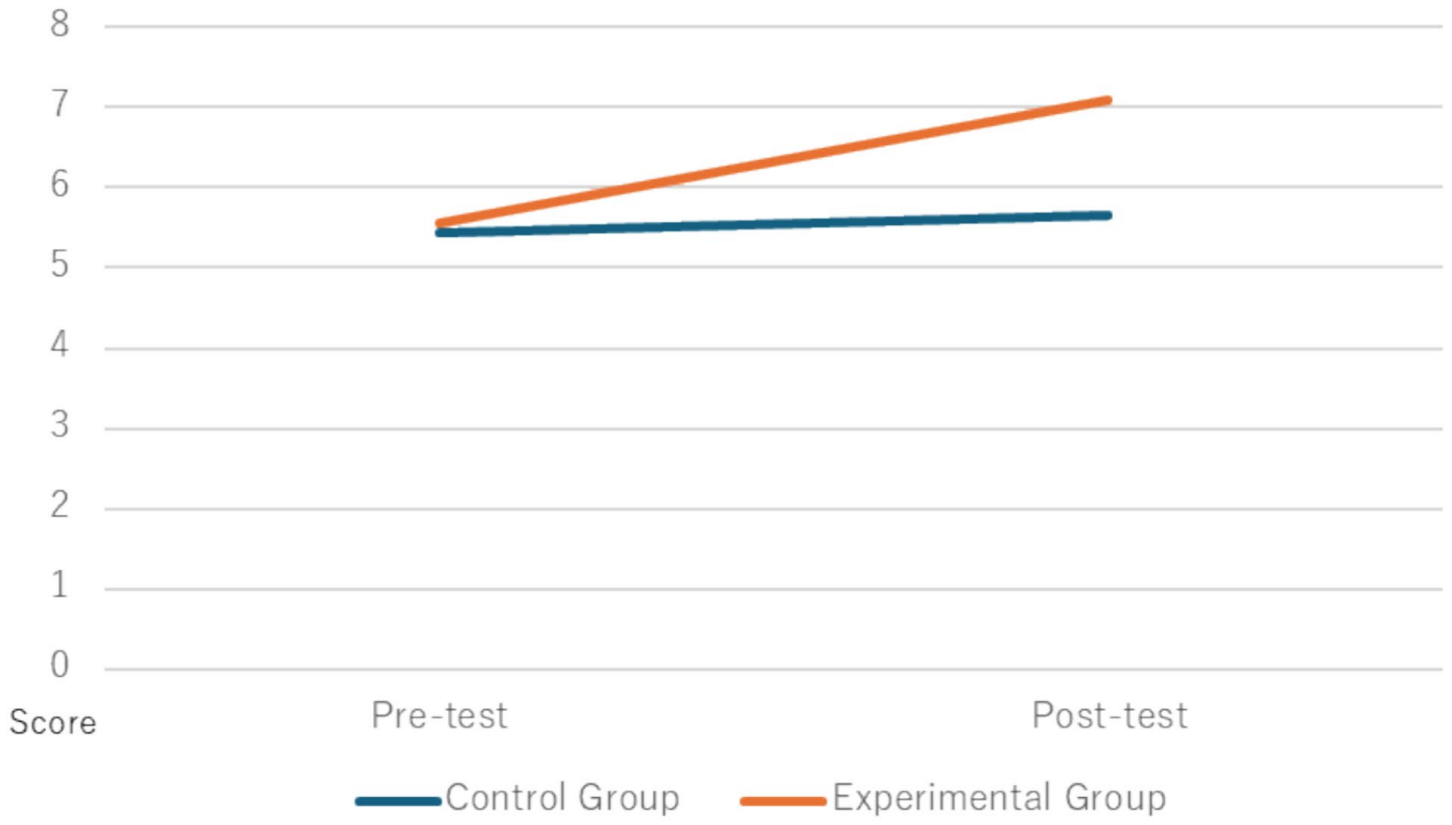
Average scores for pre- and post-tests. This figure presents the average scores of the pre- and post-tests for both the experimental and control groups. The experimental group shows a significant improvement in scores after using the training system, while the control group exhibits minimal change. These results highlight the effectiveness of the system in enhancing participants’ ability to convert first-person perspective information into a bird’s-eye view.

The scores for each group on the pre- and post-tasks are shown in Tables 3, 4. To compare the means of the pre- and post-tasks for both the control and experimental groups, a two-factor mixed-design analysis of variance (group × time: before and after using the system) was conducted. The mean scores for each group are shown in Figure 10. The main effects were tested using the Bonferroni method, and a simple main effects test was performed if the interaction was significant. Statistical analysis was conducted using IBM SPSS, with a significance level set at *p* < 0.05.

**Table 3:** Scores of the control group.

Subjects	Pre-test	Post-test
#1	6	6
#2	6	5
#3	4	6
#4	6	6
#5	6	4
#6	7	7
#7	4	6
#8	7	5
#9	6	6
#10	4	5
#11	4	6
Average	5.45	5.64

This table summarizes the pre- and post-test scores for participants in the control group. The minimal differences between pre- and post-test scores indicate that the control group, which did not use the training system, showed little to no improvement in their ability to convert first-person perspective information into a bird’s-eye view.

**Table 4:** Scores of the experimental group.

Subjects	Pre-test	Post-test
#12	5	7
#13	5	8
#14	5	7
#15	6	8
#16	6	5
#17	4	6
#18	7	7
#19	5	7
#20	5	8
#21	8	8
#22	5	7
Average	5.55	7.09

This table presents the pre- and post-test scores for participants in the experimental group. The significant increase in post-test scores demonstrates the effectiveness of the training system in enhancing the participants’ ability to reconstruct spatial relationships from a bird’s-eye perspective.

The analysis of variance showed that the interaction was significant (*F*(1, 20) = 5.11, *p* < 0.05, n^2^ = 0.21). A simple main effects test revealed that the effect before and after the intervention in the experimental group was significant (F(1, 20) = 8.56, *p* < 0.01, partial n^2^ = 0.30).

## 4 Discussion

In this study, we developed a training system aimed at converting the first-person viewpoint into a bird’s-eye perspective, and we evaluated participants’ cognitive ability to perform this conversion both before and after training. The results of the experiment showed a significant improvement in the cognitive ability to switch from a first-person to a bird’s-eye view in the group that used the training system.

In the pre- and post-tests for the control group, most subjects either maintained or lowered their scores, with only a few showing an improvement. In contrast, almost all subjects in the experimental group improved their scores, resulting in a significant difference between the two groups. Notably, as the subjects progressed through the pre- and post-tests, fewer of them made comments like “Everything looks right,” which had been common at the start of the experiment. This suggests that the participants became more adept at noticing subtle differences in the spatial arrangement of objects, particularly in discerning their front, back, left, and right orientations.

These results indicate that the system developed in this study is effective in enhancing the cognitive ability to convert from a first-person viewpoint to a bird’s-eye perspective. However, it is important to note that the ultimate goal of this research is to enhance this ability during actual ball games. In this study, we only evaluated the cognitive ability through pre- and post-tests, without testing how well participants could apply this bird’s-eye cognition while physically moving. Future research should involve field-based experiments to assess how well the system translates to real-world sports scenarios. Incorporating VR-based physical exercises, gaze tracking, or eye-tracking could simulate dynamic environments more effectively and help evaluate the practical application of the system.

Furthermore, since the training period in this study was limited to 4 days, it would be valuable to explore the effects of longer training periods. Extended training durations may reveal whether the observed improvements plateau or continue to grow over time. Additionally, future studies should include more diverse participant pools, such as athletes and individuals with varying levels of sports experience, to generalize the findings and assess the system’s utility for different populations. Despite the limited duration of this experiment, we believe that the training system developed here shows significant potential for improving the cognitive ability to convert from a first-person viewpoint to a bird’s-eye perspective.

Building on this, insights from sports psychology further underscore the importance of developing cognitive and perceptual skills to improve performance in competitive sports. Extensive research has highlighted how elite athletes excel in situational awareness and rapid decision-making during gameplay, often supported by superior visual-cognitive abilities (Mann et al., 2007; Vickers, 2007). In team sports such as soccer and basketball, where players must quickly interpret complex game scenarios, their ability to mentally switch between a first-person and a bird’s-eye view is crucial. The system developed in this study addresses this exact challenge by training players to enhance their spatial perception from multiple angles.

However, the limitations of VR environments must also be considered. While VR technology provides a controlled and immersive environment for training, it often creates spatially flat surfaces in the virtual space. Unlike real-world environments, where visual and physical cues such as uneven terrain, natural shadows, and tactile feedback aid in perceiving depth, distance, and the speed of moving objects, VR systems may lack these elements. As a result, participants’ spatial perception in VR could differ from their perception in real-world scenarios. To address these limitations, future studies should explore integrating advanced VR features, such as layered depth simulations, realistic lighting, and haptic feedback, to enhance the ecological validity of VR-based training systems.

The relationship between VR-based training and real-world performance enhancement has been increasingly highlighted in recent research. For instance, Witte et al. (2022) demonstrated that VR training significantly improved reaction behaviors in karate athletes, suggesting that such immersive environments can positively influence on-field performance. Similarly, Droste et al. (2022) found that VR not only aids in learning complex sports movements but also enhances movement quality. Proven Reality (2024) emphasized the advantages of VR for simulating game scenarios and improving decision-making under pressure. These findings align with the objectives of our study, which focuses on enhancing cognitive skills necessary for dynamic gameplay scenarios. By addressing both spatial cognition and situational judgment, the system we developed holds potential for bridging the gap between virtual and real-world performance.

In comparison to related studies like 3D-MOT, which primarily trains perceptual-cognitive abilities through object tracking tasks (Legault and Faubert, 2012; Romeas et al., 2016), our system focuses on enhancing spatial cognition by training participants to convert a first-person perspective to a bird’s-eye view. While both approaches share the goal of improving decision-making and situational awareness in dynamic environments, the methods and cognitive processes targeted differ significantly. For example, 3D-MOT emphasizes tracking and identifying specific moving objects, whereas our system requires participants to observe, memorize, and recreate spatial relationships. This distinction highlights the complementary nature of these training systems, as they address different facets of perceptual-cognitive development. Future research could explore how these methods might be integrated to provide a more holistic approach to athlete training.

Future research should incorporate field-based tests that examine whether the cognitive improvements observed in VR environments translate into better decision-making, coordination, and performance under real-world conditions. Leveraging insights from these studies, combining VR training with in-field practice could bridge the gap between virtual and actual performance enhancement, offering a more comprehensive approach to athlete development.

Previous research also suggests that experienced athletes are better able to interpret game dynamics due to their enhanced spatial cognition (Abernethy et al., 1993). For less experienced players, improving this ability to shift between different visual perspectives could be particularly beneficial. By learning to adopt a more global perspective of the field, players may be able to anticipate actions more quickly and make more accurate decisions, ultimately leading to improved game performance.

Additionally, the role of mental training in enhancing athletic performance has been widely studied. Beyond physical drills, cognitive skills such as visualization and video feedback have been shown to be effective in helping athletes sharpen their situational awareness (Williams et al., 2004). The training system we developed complements these techniques by immersing players in a realistic virtual environment, enabling them to hone their perceptual skills in a dynamic and interactive space. This could translate into better real-world decision-making during games, as athletes become accustomed to switching between first-person and bird’s-eye views more fluidly.

Looking ahead, it would be beneficial to design long-term training programs that incorporate this system. According to research in sports psychology, repeated practice combined with consistent feedback is key to improving cognitive skills (Ericsson et al., 1993). Future experiments should include both objective quantitative cognitive tests, such as reaction times and error rates, and subjective feedback to provide a more comprehensive evaluation of the system’s effectiveness. While this study primarily focused on score-based evaluations of cognitive ability, the lack of reaction time and error rate data limits our ability to fully understand the system’s impact. Incorporating these quantitative measures alongside subjective assessments in future research would enable a more nuanced understanding of the observed improvements, offering insights into their implications across different stages of training.

Moreover, modern sports psychology has also begun to focus on the collective cognitive abilities in team sports (Eccles and Tenenbaum, 2004). In team-based environments, it is not only important for individual athletes to interpret their surroundings but also for the team to share information and make coordinated decisions. There is potential to expand the current system’s application by integrating it into team training sessions, where multiple players could simultaneously practice using the bird’s-eye view to enhance collaborative decision-making. This could open new avenues for improving team dynamics and on-field coordination.

In conclusion, the training system developed in this study contributes to the growing body of knowledge on enhancing visual-cognitive skills in sports through the use of virtual reality. While short-term results are promising, future work should include field-based experiments and long-term assessments to fully understand the system’s potential in real-world sports settings. Furthermore, exploring the applicability of VR training systems in fields beyond sports, such as military operations and emergency response, could underscore the broader impact of this technology. By bridging the gap between virtual and real-world training, this study lays the groundwork for more holistic approaches to athlete development, leveraging both emerging technologies and evidence-based practices.

## 5 Conclusion

In this study, we developed and evaluated a training system designed to enhance the ability to convert visual information from a first-person perspective to a bird’s-eye view. The experimental results demonstrate that the system has potential to significantly improve cognitive abilities related to viewpoint conversion. Participants in the experimental group exhibited notable improvements in their ability to mentally shift perspectives, highlighting the system’s effectiveness.

However, while these results are promising, several limitations and areas for further investigation remain. The real-world application of this training system has not yet been tested in dynamic, game-like settings, where athletes are physically active. Future research should include field-based experiments to evaluate its impact on performance during actual gameplay. Additionally, the training period in this study was relatively short, and longer-term training experiments could provide insights into whether the observed improvements plateau or continue to increase over time.

In conclusion, this system shows great potential as a tool for enhancing athletes’ perceptual-cognitive skills, particularly in sports where quick, accurate situational awareness is crucial. Future studies in real-world sports contexts will be necessary to validate its practical benefits and explore its full potential in improving performance on the field.

## Data Availability

The datasets presented in this article are not readily available because access to the dataset is restricted to protect the privacy of participants and comply with institutional ethical guidelines. Data can only be shared with researchers upon reasonable request and approval from the ethics committee. Requests to access the datasets should be directed to kaoru.sumi@acm.org.
